# Linking adult olfactory neurogenesis to social behavior

**DOI:** 10.3389/fnins.2012.00173

**Published:** 2012-11-30

**Authors:** Claudia E. Feierstein

**Affiliations:** Vision to Action Lab, Champalimaud Neuroscience ProgrammeLisbon, Portugal

**Keywords:** olfactory neurogenesis, maternal behavior, sex discrimination, social behavior, irradiation

## Abstract

In the adult brain, new neurons are added to two brain areas: the olfactory bulb (OB) and the hippocampus. Newly-generated neurons integrate into the preexisting circuits, bringing a set of unique properties, such as increased plasticity and responsiveness to stimuli. However, the functional implications of the constant addition of these neurons remain unclear, although they are believed to be important for learning and memory. The levels of neurogenesis are regulated by a variety of environmental factors, as well as during learning, suggesting that new neurons could be important for coping with changing environmental demands. Notably, neurogenesis has been shown to be physiologically regulated in relation to reproductive behavior: neurogenesis increases in female mice upon exposure to cues of the mating partners, during pregnancy and lactation, and in male mice upon exposure to their offspring. In this scenario, and because of the key contribution of olfaction to maternal behavior, we sought to investigate the contribution of adult-generated neurons in the olfactory system to maternal behavior and offspring recognition. To do so, we selectively disrupted neurogenesis in the olfactory pathway of female mice using focal irradiation. Disruption of adult neurogenesis in the OB did not affect maternal behavior, or the ability of female mice to discriminate familiar from unfamiliar pups. However, reduction of olfactory neurogenesis resulted in abnormal social interaction of female mice, specifically with male conspecifics. Because the olfactory system is crucial for sex recognition, we suggest that the abnormal interaction with males could result from the inability to detect or discriminate male-specific odors and could therefore have implications for the recognition of potential mating partners. Here, I review the results of our study and others, and discuss their implications for our understanding of the function of adult neurogenesis.

## Adult neurogenesis

Although it was long believed that the adult mammalian brain was incapable of producing neurons, adult neurogenesis—the generation of new neurons in the adult brain—is now widely accepted to occur. Adult-generated neurons have been found in two brain areas, the olfactory bulb [OB; see **Main olfactory system (MOS)**] and the dentate gyrus (DG) of the hippocampus. However, adult-generated neurons have also been described in other brain areas, such as the hypothalamus (Kokoeva et al., 2005) and the amygdala (Fowler et al., 2008), although these results are still controversial (for a discussion see Gould, 2007; Bonfati and Peretto, 2011).

Most of our knowledge regarding adult neurogenesis comes from studies in mouse OB and DG. In both cases, neuronal precursors—the cells that have the potential to become neurons—go through a well-characterized differentiation process and give rise to neurons that will mature to become functionally integrated in the respective circuits (reviewed in Lledo et al., 2006). In the past decade or so, there has been enormous progress in characterizing the migration, differentiation and synaptic integration of new neurons into the preexisting circuits. Furthermore, we now understand some of the properties that make adult-born neurons different from neurons generated during development (reviewed in Ming and Song, 2011). However, it is still unclear how these adult-generated neurons contribute to hippocampal or olfactory function.

### Differentiation and integration of adult-generated neurons in the olfactory system

From their generation in neurogenic niches—the areas capable of producing new neurons— to their incorporation into the preexisting circuits, neuronal precursors go through a well-described series of stages (Petreanu and Alvarez-Buylla, 2002; Carleton et al., 2003). Adult-generated neurons that reach the OB originate in a layer of cells lining the lateral ventricle, the subventricular zone (SVZ) (Lois and Alvarez-Buylla, 1994). They then migrate through the rostral migratory stream (RMS), and enter the OB, where they differentiate into two different types of interneurons: granule cells (GCs) and periglomerular cells (PGCs). GCs, which constitute the majority of adult-generated neurons in the OB, form dendrodendritric synapses onto the principal cells of the OB, the mitral and tufted cells, and are thought to be important for shaping odor representations (reviewed in Urban and Arevian, 2009). Newly-generated GCs are incorporated into the existing OB circuit, and form synapses as early as 10 days after their birth (Whitman and Greer, 2007); interestingly, these first synaptic inputs come from neurons originating in cortical areas and neuromodulatory nuclei (Whitman and Greer, 2007). Later, GCs receive synapses from neurons conveying olfactory information, and are activated by odor stimuli (Carlén et al., 2002; Magavi et al., 2005). Remarkably, young neurons display unique properties: they show enhanced synaptic plasticity (Nissant et al., 2009) and increased responsiveness to odors as compared to older GCs (Magavi et al., 2005). These properties of newly-generated neurons are not exclusive to the OB, but they are also properties of adult-generated neurons in the hippocampus (Ge et al., 2007, reviewed in Deng et al., 2010).

### Regulation of neurogenesis levels

**Figure 1:**
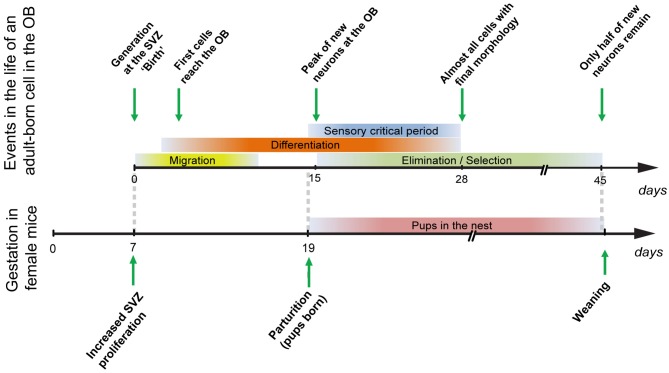
**Maturation, differentiation, and selection of adult-generated OB cells (top) in comparison to the events occurring during gestation in female mice.** Cells that are generated during gestation (day 7; Shingo et al., 2003) will reach the OB and enter their critical period at the time of parturition, when mothers first encounter their pups (see main text).

The levels of adult neurogenesis are regulated by a variety of environmental factors, which can influence the proliferation of precursors in the neurogenic niches, neuronal survival, or both. Of the tens of thousands of new neurons that reach the mouse OB every day (Alvarez-Buylla and Garcia-Verdugo, 2002), only a fraction survives: the number of new neurons present in the OB peaks at 15 days (Figure 1) after their generation in the SVZ; new neurons then undergo an elimination/selection process (Figure 1), so that only one half of a given cohort of adult-generated neurons remains 45 days after their generation.

Adult neurogenesis levels depend on olfactory activity. Whereas olfactory deprivation decreases survival of newly-generated neurons (Mandairon et al., 2003; Yamaguchi and Mori, 2005), particularly during a critical period 14–28 days after their birth (Figure 1), olfactory enrichment increases their survival (Petreanu and Alvarez-Buylla, 2002; Rochefort and Lledo, 2005; Mandairon et al., 2006b). In the hippocampus, neurogenesis increases with environmental enrichment, exercise and hippocampal-dependent tasks. On the other hand, neurogenesis levels are reduced by social isolation and stress (Lu et al., 2003; Kannangara et al., 2009).

Learning has also been shown to affect the levels of neurogenesis: both perceptual and associative olfactory learning result in a selective recruitment of new neurons to areas that are activated by the odors learned (Alonso et al., 2006; Mandairon et al., 2006a; Moreno et al., 2009; Sultan et al., 2010). Interestingly, this effect of learning depends on the age of the neurons: while olfactory training increases survival of neurons between 18 and 30 days of age, it decreases survival of neurons aged 38 days (Mouret et al., 2008). Similar age-dependent survival has been observed in the accessory olfactory bulb [AOB; see **Accessory olfactory system (AOS)**] of female mice exposed to male **pheromones** (Oboti et al., 2011).

### Functional contribution of adult-generated neurons

How do adult-generated neurons contribute to olfactory or hippocampal function? Several studies have attempted to address this question by altering neurogenesis levels and asking how behavior is affected. Although those experiments hint to a role of adult neurogenesis in learning and memory, the results remain inconclusive. Different approaches have been used to manipulate the levels of neurogenesis, and those approaches affect neurogenesis with different specificity and to a different extent (see section “Experimental Disruption of Olfactory Neurogenesis”); as a consequence, they could produce varying effects on olfactory behavior. In addition, a variety of behavioral protocols have been used.

Decreasing neurogenesis levels results in seemingly conflicting observations. While some studies reported impaired short-term odor memory but intact long-term odor memory when neurogenesis was disrupted (Breton-Provencher et al., 2009), others reported the opposite (Lazarini et al., 2009; Sultan et al., 2010). As for odor discrimination ability, disruption of adult neurogenesis has been reported to alter discrimination in a spontaneous discrimination test (Moreno et al., 2009), but other studies observed intact discrimination ability in both spontaneous and learned discriminations (Imayoshi et al., 2008; Breton-Provencher et al., 2009; Lazarini et al., 2009). Fewer studies have examined the effects of increasing neurogenesis on behavior. Among them, it was shown that increasing neurogenesis by exposing mice to an odor-enriched environment results in longer-lasting odor memory (Rochefort et al., 2002). All in all, despite the contradictory results, an emergent feature of all these studies is the implication of adult-generated neurons in learning and memory processes (similarly in the hippocampus, see below).

Experimental manipulations that alter the levels of neurogenesis could provide useful insight to understanding the contribution of adult neurogenesis. Nevertheless, if there are situations when neurogenesis is *physiologically* regulated, these could prove more informative to revealing the role of adult-generated neurons: it is tempting to assume that, if neurogenesis is naturally modulated in a given context, new neurons are likely to be important to that process. Regulation of adult neurogenesis levels in physiological situations has been described in the context of reproductive behaviors. These behaviors rely heavily on olfactory cues: anosmic mice cannot distinguish normal from castrated males (Lin et al., 2005; Keller et al., 2006) and show impaired mating behavior (Vandenbergh, 1973). Furthermore, OB lesions abolish the establishment of maternal behavior (Gandelman et al., 1971). It is interesting, therefore, that neurogenesis is modulated in the olfactory system (and in the hippocampus) in situations associated to reproductive behavior: in female mice and rats, neurogenesis increases in the OB during pregnancy and lactation (Shingo et al., 2003), (Furuta and Bridges, 2005), and upon exposure to male pheromones in both the main OB and AOB (Mak et al., 2007; Larsen et al., 2008; Oboti et al., 2009), and in male mice upon interaction with their offspring (Mak and Weiss, 2010). Contrary to rodents, a downregulation of neurogenesis has been reported in the olfactory system of sheep during parturition and interaction with the newborns (Brus et al., 2010). Thus, exploring the contribution of adult-generated neurons to reproductive behaviors seems a promising avenue to understanding their function.

In this context, in a recent study (Feierstein et al., 2010), we asked whether disrupting olfactory neurogenesis would affect reproductive behaviors, in particular, maternal behavior. We found that substantial elimination of adult-generated neurons in the OB did not result in impaired maternal behavior or offspring recognition, but rather in defects in social interaction (Feierstein et al., 2010). In particular, female—male interactions were abnormal, suggesting that adult-generated neurons could be important for sexual recognition. Here, I review the results of that study, and contrast them with other studies investigating the role of adult neurogenesis, hoping to provide a comprehensive view that may help us understand how adult neurogenesis contributes to brain function.

## Is adult olfactory neurogenesis important for social and reproductive behaviors?

### Olfaction and maternal behavior

Given the crucial role of olfaction in maternal behavior (Gandelman et al., 1971), several findings pieced together suggested to us that olfactory neurogenesis could also be important for the establishment or expression of maternal behavior. First, neurogenesis had been shown to increase during pregnancy and lactation (Shingo et al., 2003; Furuta and Bridges, 2005). In mice, this increase in neuronal proliferation occurs at day seven of gestation (Figure 1). Because pregnancy in mice lasts 19–20 days, and because newly-generated GCs enter a critical period for activity-regulated survival at around 2 weeks of age (Yamaguchi and Mori, 2005), neurons generated during pregnancy would arrive at the OB and enter their critical period around the time of parturition (Figure 1), suggesting that they could be implicated in learning pup odors. Second, both exposure of female rats to male pheromones or their treatment with prolactin (PRL)—a hormone essential to the changes induced by pregnancy that lead to proper maternal care (Mann and Bridges, 2001)—result in a concomitant increase in olfactory neurogenesis and an advancement of maternal behavior (Larsen et al., 2008).

To investigate the contribution of adult-generated neurons in the olfactory system to maternal behavior and pup recognition, we disrupted adult neurogenesis in the OB of female mice, and asked how this manipulation affected behavior. Two factors are crucial when asking this question and interpreting the results of our study and others: the strategy used to alter neurogenesis, and careful and detailed analysis of this complex behavior.

### Experimental disruption of olfactory neurogenesis

Current methods for manipulating neurogenesis are rather nonspecific. Three different approaches are used to disrupt neurogenesis, each with its own advantages and disadvantages: antimitotic drugs, genetically-targeted ablation, and irradiation. The use of antimitotic drugs can provide temporal specificity, as neurogenesis is blocked only while the drug is administered (Doetsch et al., 1999; Wei et al., 2011). Moreover, an almost complete ablation of newly-generated neurons can be achieved with these drugs. However, although toxicity can be avoided when using low doses of these drugs, the main problem with this approach is the lack of spatial specificity: infusion of antimitotic drugs affects not only olfactory neurogenesis, but also hippocampal neurogenesis (Mak et al., 2007). Genetically-targeted ablation, to date, suffers as well from the lack of specificity for targeting different neurogenic niches, disrupting *both* hippocampal and olfactory neurogenesis (Imayoshi et al., 2008; Sakamoto et al., 2011), and other potential neurogenic sites (Gould, 2007; Bonfati and Peretto, 2011). Thus, for these two approaches, it is difficult, if not impossible, to dissociate the contribution of each system to behavior. On the other hand, irradiation can be used to disrupt cell proliferation in a more localized manner, targeting specifically the SVZ (Lazarini et al., 2009; Valley et al., 2009) or the hippocampus (Santarelli et al., 2003) to impair olfactory or hippocampal neurogenesis, respectively; on the downside, irradiation results in a chronic and often incomplete ablation of neural precursors.

**Figure 2:**
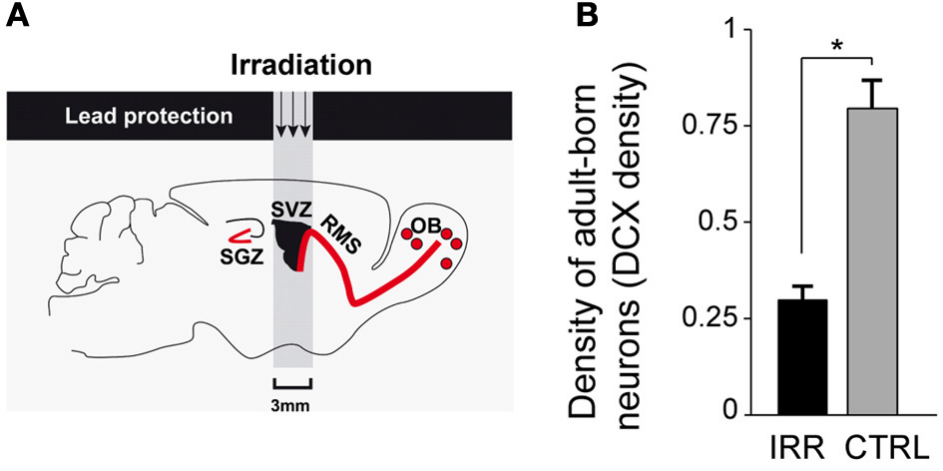
**Disruption of adult olfactory neurogenesis. (A)** Gamma irradiation was targeted to the SVZ, protecting the rest of the brain with a lead shield (for details see Lazarini et al., 2009; Feierstein et al., 2010). The SGZ, the site of hippocampal neurogenesis, was not affected. **(B)** Adult neurogenesis was reduced in the OB of irradiated (IRR) compared to control (CTRL) females. Density of adult-born neurons is measured as the density of DCX^+^ (a marker expressed in immature neurons) cells in the OB 6.5 months after irradiation. ^*^p < 0.01. OB, olfactory bulb; SVZ, subventricular zone; RMS, rostral migratory stream; SGZ, subgranular zone. Modified from Feierstein et al. (2010).

To disrupt neurogenesis *specifically* in the OB and to avoid the confounds of a more generalized blockade, we used focal gamma irradiation of the SVZ (Figure 2A) of 8-week-old virgin female mice (Feierstein et al., 2010), which leaves hippocampal neurogenesis unaffected (Lazarini et al., 2009). Having established that gamma irradiation resulted in a substantial, chronic, reduction of adult-generated neurons reaching the OB (Figure 2B), we went on to test the effects of this treatment on a range of social and reproductive behaviors.

### Does impaired neurogenesis affect maternal behavior?

Studies evaluating maternal behavior focus on the behavior at the nest and the interaction of females with the pups in the home-cage environment (time spent in a nursing posture, time grooming and licking pups), as well as retrieval of pups to the nest when they are dispersed (Myers et al., 1989; Lucas et al., 1998; Brown et al., 1999; Lonstein and Fleming, 2001). Several of these behaviors are indicative of the level of engagement in maternal care and influence the development of the litter and their adult behavior (Myers et al., 1989; Champagne et al., 2003). The establishment and expression of maternal behavior require the coordinated action of neural (Slotnick and Nigrosh, 1975) and hormonal factors (Bridges et al., 1985; Lucas et al., 1998), involved, amongst others, in processing sensory cues from the offspring and providing the attachment and motivation to care for the pups (Kendrick et al., 1997). Thus, impairments in maternal behavior can result from disrupting olfactory processing (Gandelman et al., 1971), but also from altering the hormonal changes that accompany pregnancy, or affecting motivational systems or stress and anxiety levels (Yamada et al., 2002; Coutellier et al., 2009; Kessler et al., 2011).

**Figure 3:**
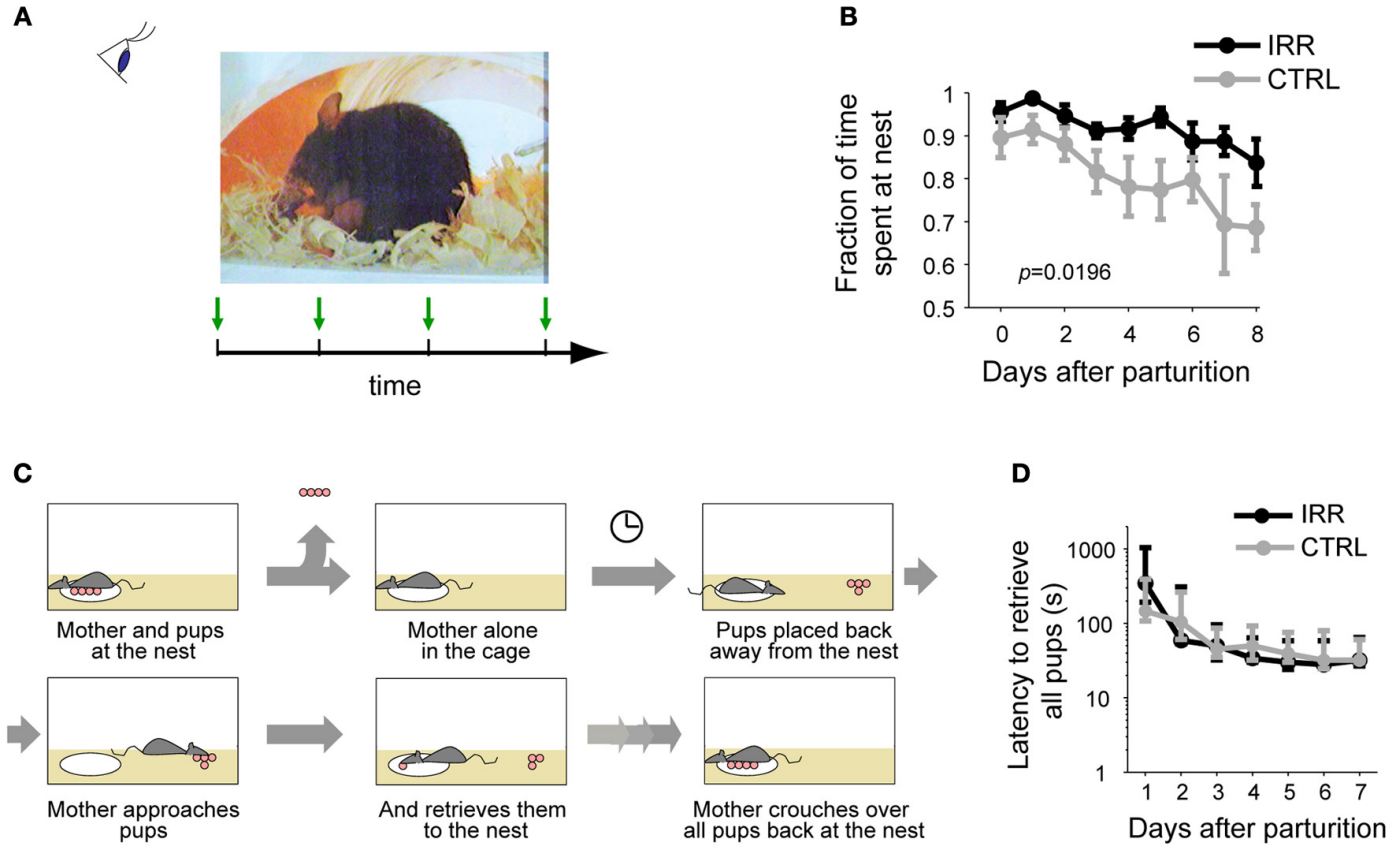
**Maternal behaviors evaluated. (A)** Maternal behavior at the home-cage was observed and scored periodically. **(B)** Fraction of time (of the total observation time) the females spent at the nest with the pups across days after pup birth. Data are represented as the mean ± SEM across mice in each treatment. IRR females stayed longer at the nest than CTRL females (Feierstein et al., 2010). **(C)** Schematic of pup retrieval. Retrieval behavior was tested daily, starting the day after parturition (P1). **(D)** Latency to finish retrieval (the fourth pup brought back to the nest) was identical in CTRL and IRR females. Data are shown as median latencies, in logarithmic scale; error bars represent 25th and 75th percentiles.

We evaluated the maternal behavior in the home-cage of both treated (IRR: irradiated) and control (CTRL) females (Figure 3A). First, we compared the time spent at the nest with the pups, and observed that IRR females spent a slightly, but significantly, larger amount of time at the nest with the pups (Figure 3B; Feierstein et al., 2010). As a result, IRR mothers tended to spend a bit more time feeding their litters; however, this did not result in differences in the development of the pups (Feierstein et al., 2010). All other home-cage behaviors evaluated were indistinguishable between CTRL and treated mothers (Feierstein et al., 2010).

We then tested pup retrieval (Figure 3C). In our hands, both IRR and CTRL females retrieved pups to the nest, and their behavior was identical both in terms of the latency to bring the pups back to the nest, and the decreased times to perform this behavior across days (Figure 3D; Feierstein et al., 2010).

Thus, selective disruption of olfactory neurogenesis using irradiation left maternal behavior unaffected. In a recent study Larsen and Grattan (2010) blocked neurogenesis using a subcutaneous injection of an antimitotic agent (blocking neurogenesis both in the olfactory system and the hippocampus); similar to our findings, they observed that pup retrieval was unaffected when females were tested in their home-cage. However, Larsen and Grattan (2010) observed that pup retrieval was impaired when females were tested in a novel cage. This result could be attributed to increased anxiety levels as a consequence of blocking neurogenesis in the hippocampus, and could be further enhanced by the stress generated by the test (Snyder et al., 2011). Similarly, a recent study by Sakamoto et al. (2011) claims that pup retrieval is disrupted in mice with a genetic ablation of forebrain neurogenesis; unfortunately, they do not show the data to support this claim; moreover, the blockade of neurogenesis used was not brain-area specific, making it impossible to assess the contribution of olfactory neurogenesis to any of the behaviors tested in their study.

Neither of the studies mentioned above tested maternal behavior in the nest (how much time females spend in the nest, nursing, grooming of pups); to my knowledge, our study is the only one were undisturbed maternal care at the home-cage was evaluated. Moreover, it is important to note that most, if not all, studies of maternal behavior have been done in animals were blockade of neurogenesis was not specific to the olfactory system (see above; Larsen and Grattan, 2010; Sakamoto et al., 2011; Wei et al., 2011). This is problematic not only because it is impossible to dissociate the potential contributions of hippocampal and olfactory neurogenesis, but also special caution should be taken when evaluating any hormonally-regulated behavior, given that neurogenesis has also been reported in the hypothalamus (Kokoeva et al., 2005), and affecting this structure is likely to perturb reproduction-related behaviors. One possible caveat of our irradiation protocol is that the irradiation could have affected the anteroventral periventricular (AVPv) nucleus, located at the floor of the brain within the window we irradiated. This nucleus is important for regulating reproductive function, in particular the activity of gonadotropin-releasing hormone (GnRH) neurons (Gu and Simerly, 1997; Semaan and Kauffman, 2010). Although neurogenesis has been described in this nucleus during the prepurbertal period in rats (Ahmed et al., 2008), it is unknown whether neurogenesis continues into adulthood (the time at which we performed the irradiation). In our hands, irradiation of the SVZ did not affect reproductive function: IRR females mated normally, carried normal pregnancies, and lactated normally; therefore, it is unlikely that our manipulation disrupted AVPv function.

Thus, when neurogenesis is impaired in the olfactory system without affecting the hippocampus, and in the absence of confounding variables such as changes in anxiety levels or stress induced by the exposure to a novel environment, both maternal care at the home-cage and pup retrieval are unaffected (Feierstein et al., 2010).

### Is olfactory neurogenesis required for pup recognition?

The results summarized above suggest that adult-generated neurons are not required for the establishment or expression of maternal behavior (Feierstein et al., 2010), which may be explained by the fact that the behaviors tested are likely to rely on the motivational drive to behave *maternally*, and are perhaps not sensitive to partial disruption of olfactory function. It is possible, however, that new neurons are important for learning the odors of the progeny as *own*, and to distinguish the own progeny from unrelated individuals.

The ability to recognize offspring seems to be an important component of the maternal experience. In species such as sheep, odor learning is particularly important for forming the maternal bond: ewes need to interact with their cubs to learn their smell, and will reject lambs that they do not recognize as their own (Brennan and Kendrick, 2006; Lévy and Keller, 2009). Mice seem to use kin recognition both for nesting and mating: while they will form communal nests in the wild, they will do so preferentially with related individuals (Manning et al., 1992). As for mating, mice will choose their mating partners according to **relatedness** (Barnard and Fitzsimons, 1988; Potts et al., 1991); importantly, this relatedness is not merely based on genetic similarity or self-inspection, but seems to be learned: female mice are less likely to mate with cross-fostered individuals, even if less genetically similar (Penn and Potts, 1998a), suggesting that they learn the odors of the conspecifics they share the nest with as “*related* individuals.”

Nesting and mating preferences have been demonstrated in the wild and in seminatural conditions; however, laboratory strains have low or no genetic variability. Can laboratory mice discriminate their young amongst others? In laboratory settings, **outbred mice** are able to distinguish their pups from others (Ostermeyer and Elwood, 1983), and even discriminate individuals differing in a single MHC locus (Penn and Potts, 1998b; Yamazaki et al., 2000).

**Figure 4:**
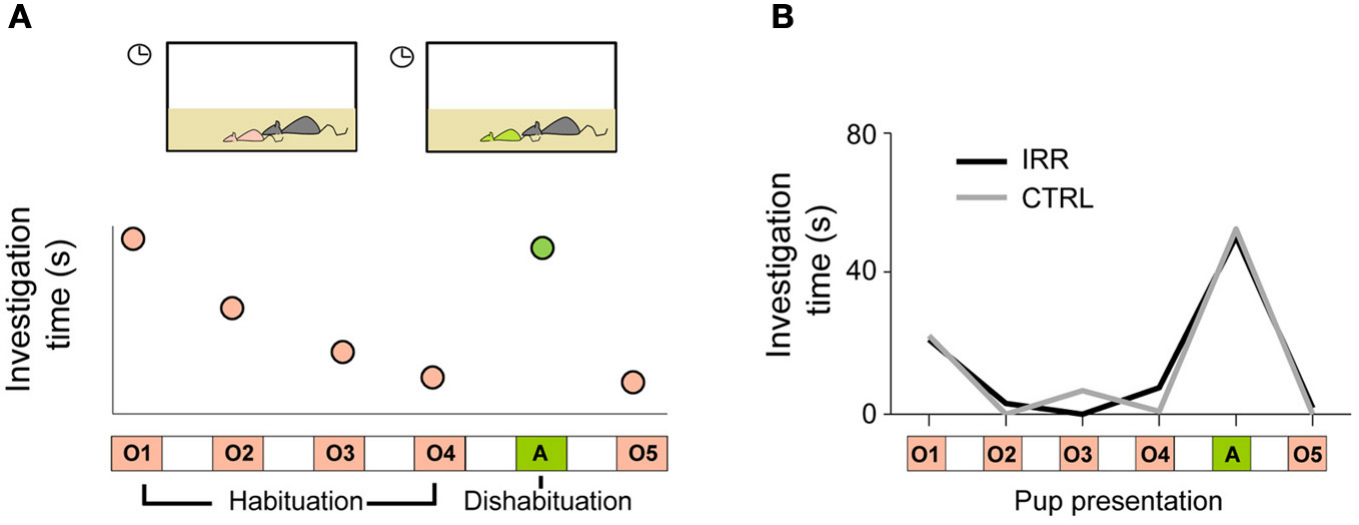
**(A)** Schematic of the habituation/dishabituation protocol for testing pup discrimination. Repeated presentation of a mother's own pup (O1 to O4) results in decreased investigation (measured as sniffing and grooming) time. When a pup from a different litter is presented (A: alien), investigation time increases—this shows that the tested subject can discriminate between the pups. **(B)** Both IRR and CTRL females discriminated own pups vs. an alien one (compare O4 to A; *p* < 0.05). Investigation time is shown as the median for each treatment.

Offspring recognition by mothers had not been shown for **inbred mouse** strains (Mak and Weiss, 2010), so we first asked whether female mice (of the inbred strain C57Bl6) were able to discriminate their pups from others, and if so, whether adult-generated neurons would play a role in this ability. To address these questions, we used a habituation/dishabituation paradigm (Figure 4A; Ostermeyer and Elwood, 1983); in this type of paradigm, animals show that they recognize a subject (or more generally an odor or object) by an increase in investigation when a novel subject is presented (Winslow, 2003). Females showed increased investigation time when an unfamiliar pup was presented, compared to investigation of a pup from their offspring (Figure 4B), showing that inbred mice can discriminate amongst individuals. However, IRR mice showed identical ability to discriminate between **familiar** and unfamiliar pups (Figure 4B), demonstrating that disruption of adult olfactory neurogenesis does not impair the ability to discriminate between subjects (Feierstein et al., 2010). It is important to note that, even though females can discriminate a familiar pup from an unfamiliar one, this does not imply they *recognize* the pups as own or related.

### Is olfactory neurogenesis important for social interaction?

The above result shows that mice are able to distinguish a familiar from an unfamiliar pup. What would this type of ability be useful for? As discussed above, being able to discriminate own vs. alien progeny could serve two important purposes: first, to provide selective care to one's own progeny; second, to avoid mating with one's own progeny (inbreeding). Mice have been shown to display mating preferences according to the degree of relatedness (Barnard and Fitzsimons, 1988; Potts et al., 1991). Notably, this preference is different for males and females: while males prefer to mate with more genetically-distant females, females show preference for closely-related male siblings or half-siblings (Barnard and Fitzsimons, 1988).

We therefore tested whether female mice would show differential interaction with own vs. unrelated juveniles (given that they can discriminate them) or adults. Shortly after pup weaning (juveniles age 22 days), both IRR and CTRL females were presented simultaneously with a mouse of their own litter and one from a different litter. Neither CTRL nor treated females displayed a preference in the interaction with own vs. alien juveniles; intriguingly, IRR females spent twice as long in contact with the juveniles when compared to CTRL females.

Given that females did not show a preferential investigation of own vs. alien juveniles, we wondered whether a preference for interacting with own progeny vs. other mice would develop later, when juveniles became sexually mature. Again, neither CTRL nor IRR females showed a differential interaction with mice from their own litter vs. unrelated mice. This is in contrast with a recent study on paternal behavior (Mak and Weiss, 2010), where male mice displayed differential investigation of offspring vs. nonoffspring in a similar paradigm. It remains unclear whether the difference between that study and ours arises from behavioral differences between genders. This possibility is intriguing, given that females prefer to mate with siblings or half-siblings (Barnard and Fitzsimons, 1988), whereas males preferentially choose less-related individuals, and this could be reflected in the preferential interaction of males with nonoffspring.

**Figure 5:**
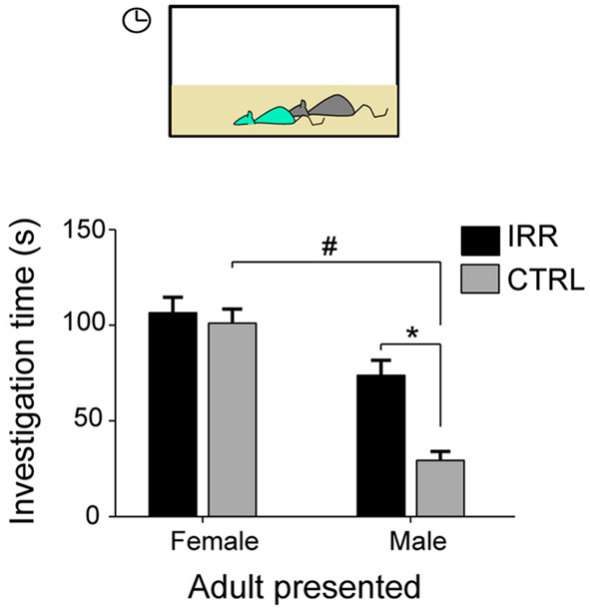
**Social interaction is altered in IRR females.** Control females showed differential interaction with male and female conspecifics (#). However, IRR females behaved similarly toward both genders: interaction with male subjects resembled that observed with female subjects. Investigation time is shown as mean across experimental females. ^*^Significant difference between CTRL and IRR for the interaction with males. ^#^Significant difference for CTRL females for the interaction with adult males and females. Modified from Feierstein et al. (2010).

Thus, disruption of olfactory neurogenesis in females did not result in changes in their ability to discriminate familiar vs. unfamiliar juveniles and adult mice, nor did it affect females' preferences to interact with either of them. Notably, however, IRR females showed an altered patterned of social interactions which resulted from a differential behavior toward different genders (Figure 5): while CTRL females interacted differently with adult male and female subjects, IRR females showed the same interaction with adult subjects of either sex (Feierstein et al., 2010). One possible interpretation is that IRR females failed to detect male odors. In agreement with this hypothesis, olfactory neurogenesis is necessary for the establishment of a preference for dominant males (Mak et al., 2007). It is noteworthy that, although IRR females showed an altered social interaction pattern, they did not show differences in the investigation of urine odors from conspecifics (Feierstein et al., 2010), suggesting that other odorants/pheromones not present in the urine are important for sex recognition, and this detection is altered in IRR females. Male recognition by females mice is mediated by a variety of substances, secreted into urine and other body secretions such as tears, and acting both on the MOS and AOS (Hurst, 2009; Baum, 2012). These systems are thought to play complementary roles in mate recognition, although their unique contribution remains a matter of debate. An emerging view is that while the MOS mediates the detection of maleness signals and approach to investigate males (Baum, 2012), the AOS is involved in extracting more detailed information such as individuality signatures, social and health status (Hurst, 2009), and inducing reproductive behaviors such as lordosis (Haga et al., 2010). In our study, IRR females seem to fail to recognize males as such, based on the interaction patterns observed, suggesting that detection of male-specific cues is impaired due to disrupted neurogenesis. It would be interesting to investigate whether this alteration in the interaction patterns is reflected on the mating behavior of these females. In addition, it would be important to establish whether neuronal responses to male cues are disrupted in IRR females.

## Adult neurogenesis, olfactory memories and behavior: problems and emerging principles

### Limitations of this and other studies

Despite the increasing number of studies trying to address the role of neurogenesis in olfactory function, a clear understanding of its function remains elusive. This can be attributed, at least partly, to several methodological problems.

First, genetic and pharmacological methods for manipulating neurogenesis are rather nonspecific (see section “Experimental Disruption of Olfactory Neurogenesis”). An important strength of our study is that focal irradiation of the SVZ resulted in disruption of olfactory neurogenesis, sparing the hippocampus. In this way, we eliminated the possibility of attributing the observed deficits to altered hippocampal function, a confound present in most studies to date (i.e., Larsen and Grattan, 2010; Sakamoto et al., 2011). However, although disruption of neurogenesis was rather specific, it was both chronic and not complete. Therefore, the lack of effect observed in the behaviors tested could be attributed to compensatory mechanisms that become active when neurogenesis is disrupted, or to the fact that some neurogenesis remains after the treatment. Thus, it is important to take into account the compromise between the degree of neurogenesis disruption and its spatial and temporal specificities. More precise methods for manipulating neurogenesis or the activity of the new neurons will help elucidate their contribution to brain function.

Second, behavioral responses such as stress responses, anxiety and maternal care, differ not only between species, but also across mouse strains, and the contribution of adult-generated neurons to behavior could also differ. Importantly, the levels of adult neurogenesis and its regulation differ across rodent species, particularly between wild and laboratory mice (Amrein et al., 2004; Klaus et al., 2012): while environmental enrichment and physical activity increase neurogenesis in laboratory mice, this effect is less pronounced in wild mice (Klaus et al., 2012). Thus, it is possible that the difficulty in pinning down the contribution of adult-generated neurons to behavior arises from studying mice in laboratory conditions, where they live in a restricted, stimulus-poor environment.

Third, neurogenesis levels decrease with age (Amrein et al., 2004; Enwere et al., 2004; Luo et al., 2006). Thus, it is important to consider the age at which neurogenesis is manipulated: for instance, at an old age, when neurogenesis levels are low, increasing neurogenesis through environmental enrichment could have stronger impact on behavior than in younger animals; conversely, ablating neurogenesis at an old age could result in negligible effects on behavior. In addition, neurogenesis could have different roles in juvenile vs. adult mice: while ablating neurogenesis in juvenile mice results in impairments in social behavior (Wei et al., 2011), the same treatment in adult mice does not (Wei et al., 2011). In our study, different behaviors were tested at different ages (Feierstein et al., 2010); it is therefore possible that lower neurogenesis levels at an older age resulted in a stronger effect on behavior.

Finally, the behavioral protocols and training used in different studies are highly variable, and behavioral analyses often superficial (this is particularly true for the analysis of social behavior), making it difficult to compare across studies and to draw unifying conclusions. For instance, the different effects on learning and memory observed when neurogenesis is disrupted could be attributed to testing of behaviors that invoke different learning mechanisms, such as perceptual vs. reward-based learning (Lazarini and Lledo, 2011; Breton-Provencher and Saghatelyan, 2012). More careful behavioral analyses and consideration of the variables that influence the behaviors tested will be crucial for a better understanding of the contribution of adult-generated neurons to behavior.

### Adult-generated neurons and distinct neuronal representations

So what do adult-generated neurons do after all? Despite the contradictory effects on behavior that result from manipulating neurogenesis (Deng et al., 2010; Lazarini and Lledo, 2011; Breton-Provencher and Saghatelyan, 2012), a general principle seems to emerge from the effects of these manipulations: that adult-generated neurons are important for learning and memory (Deng et al., 2010; Lazarini and Lledo, 2011; Breton-Provencher and Saghatelyan, 2012). But how do they contribute to these processes? Computational models suggest that the constant addition of neurons aids in discrimination, particularly when the items (environments and stimuli) to discriminate are very similar, and they would do so by making the representation of different items more distinct (Cecchi et al., 2001; Aimone et al., 2011; Sahay et al., 2011b). Adult-generated neurons provide a constant pool of cells with unique properties—enhanced synaptic plasticity and increased responsiveness to stimuli—that could contribute to forming more distinct stimuli representation. This could be achieved at the level of encoding the stimulus by providing nonoverlapping populations that are activated by different stimuli (Sahay et al., 2011b), or by creating “high-resolution” representations that can be more easily retrieved (Aimone et al., 2011). In either case, more separate representations of stimuli or events would be beneficial for learning and memory. Indeed, neurogenesis in the DG has been shown to be important for spatial discrimination of similar contexts (Clelland et al., 2009; Tronel et al., 2010; Sahay et al., 2011a), suggesting that adult-born neurons aid to the pattern separation function of the DG.

As for olfaction, whether adult-generated neurons contribute to odor memory or odor discrimination remains controversial (see section “Functional Contribution of Adult-Generated Neurons”). Notably, in a recent study, Alonso et al. (2012) showed that specific activation of adult-born neurons (although mature ones) accelerated olfactory learning when using similar odorants, leaving discriminability intact; moreover, behavioral changes were accompanied by increased inhibition of principal neurons in the OB, which is believed to result in more distinct odor representations (Urban and Arevian, 2009).

### Link to social and reproductive behavior: learning of social odors

What have we learned about the role of olfactory neurogenesis in social and reproductive behavior? Olfactory neurogenesis is regulated *physiologically* in several instances associated to reproduction: during pregnancy and lactation (Shingo et al., 2003), upon exposure to opposite-sex pheromones (Mak et al., 2007; Larsen et al., 2008; Oboti et al., 2009), or interaction of male mice with their offspring (Mak and Weiss, 2010). It is tempting to speculate therefore that new neurons added to the olfactory system contribute to odor learning or odor memory associated to those instances: learning the odor of the mating partner—crucial to avoiding **pregnancy-block (or Bruce effect)** (Bruce, 1959)—, or learning the odor of the offspring, important for providing selective care and avoiding inbreeding (Pusey and Wolf, 1996; Penn and Potts, 1998a; Sherborne et al., 2007).

A few recent studies, including ours (Feierstein et al., 2010), have attempted to establish a link between neurogenesis and reproductive behaviors. In our hands, disruption of olfactory neurogenesis had no effect on maternal care, or the ability of female mice to discriminate pups, suggesting that new neurons were not necessary to perform such a discrimination (this should be interpreted with caution, given that ablation of neurogenesis was not complete). Although we hypothesized that female mice would interact differentially with juveniles of their own vs. other litters, we did not observe such a difference. In a recent study, however, Mak and Weiss (2010) observed that male mice investigated differentially familiar vs. unfamiliar juveniles, and this differential investigation depended both on intact neurogenesis and previous exposure to the pups, suggesting that adult-born neurons played a role in learning and forming a memory of the pups odors. Nevertheless, in that study neurogenesis was also disrupted in the hippocampus, making it difficult to determine whether the deficit depended on neurogenesis in the hippocampus or the OB (or both). Unfortunately, because our study and that of Mak and Weiss (2010) tested offspring recognition in different genders, it is difficult to directly compare the results, and the differences could be attributed to different behavior in males and females.

The MOS and AOS have complementary roles in mating and reproductive behavior (Baum and Kelliher, 2009), and both are recipient of new neurons in the adult. The MOS is believed to be involved in sexual recognition (Baum, 2012); our results suggesting a deficit in sex recognition would be consistent with an involvement of the MOS in this function (Feierstein et al., 2010). On the other hand, it has been suggested that the AOS is important for the recognition of *specific* mating partners (Hurst, 2009), and therefore important for the induction or prevention of pregnancy-block. In this context, it is interesting to consider a recent study by Oboti et al. (2011), where they showed that exposure to male pheromones increases the survival of neurons in the AOB. Notably, intact adult neurogenesis at the time of mating was important for the prevention of pregnancy-block, suggesting that adult-generated neurons participated in forming the memory of the mating partner (Oboti et al., 2011). Thus, studies of neurogenesis and social behavior suggest that adult-generated neurons contribute to forming relevant social memories, such as that of the mating partner or the progeny. Functional experiments are needed to evaluate the contribution of adult-generated neurons to the representation of social odors.

### Conflict of interest statement

The author declares that the research was conducted in the absence of any commercial or financial relationships that could be construed as a potential conflict of interest.

## Key concept

Main olfactory system (MOS): Olfactory structures implicated in the detection of nonvolatile (and some volatile) odors. Olfactory receptor neurons in the main olfactory epithelium (MOE) project to the main olfactory bulb (MOB), which then sends projections to cortical and subcortical structures.
Accessory olfactory system (AOS): Olfactory structures associated with the detection of pheromones and volatile compounds. Odorants are detected by sensory neurons in the vomeronasal organ (VNO). These neurons project to the accessory olfactory bulb (AOB), which in turn sends projections to subcortical areas implicated in emotional and social behavior.
Pheromones: A chemical signal that is secreted by an organism and is capable of eliciting behavioral or physiological responses in other individuals. Sex pheromones, for instance, can induce hormonal responses, approach behavior and therefore facilitate reproduction.
Relatedness: A *related* individual is one that is genetically linked (for instance, a sibling). Relatedness can also be related to being raised together (see main text).
Outbred mice: Laboratory mice that, although genetically similar, show greater genetic variability than inbred mice.
Inbred mice: Strains with little or no genetic variability, which result from prolonged incrossing (inbreeding).
Familiar: A *familiar* individual is one that has been encountered before.
Pregnancy-block (or Bruce effect): Termination of pregnancy that occurs during the first days of gestation if female mice are exposed to the odors of unfamiliar males, but not to those of the mating partner.
